# Supplementary material for: HIVprotI: an integrated web based platform for prediction and design of HIV proteins inhibitors
Source: J Cheminform. 2018 Mar 9;10:12. doi: 10.1186/s13321-018-0266-y (PMC5845081; doi:10.1186/s13321-018-0266-y)
Supplement: Supplementary file 2 — Additional file 2. Source code of HIVProtI web server. [file 13321_2018_266_MOESM2_ESM.zip › HIVprotI_Source-code/hivprotI/weka-3-6-6/doc/weka/filters/unsupervised/attribute/MakeIndicator.html]

MakeIndicator


---


|  |  |  |
| --- | --- | --- |
| **Overview** \| **Package** \| **Class** \| **Tree** \| **Deprecated** \| **Index** \| **Help** \| **Weka's home** | |  |
| **PREV CLASS**   **NEXT CLASS** | **FRAMES**    **NO FRAMES**     **All Classes** |  |
| SUMMARY: NESTED \| FIELD \| CONSTR \| METHOD | DETAIL: FIELD \| CONSTR \| METHOD |  |


---


## weka.filters.unsupervised.attribute Class MakeIndicator

```
java.lang.Object
  weka.filters.Filter
      weka.filters.unsupervised.attribute.MakeIndicator
```

**All Implemented Interfaces:**: java.io.Serializable, CapabilitiesHandler, OptionHandler, RevisionHandler, StreamableFilter, UnsupervisedFilter

---

``` public class MakeIndicator extends Filter implements UnsupervisedFilter, StreamableFilter, OptionHandler ```

A filter that creates a new dataset with a boolean attribute replacing a nominal attribute. In the new dataset, a value of 1 is assigned to an instance that exhibits a particular range of attribute values, a 0 to an instance that doesn't. The boolean attribute is coded as numeric by default.


Valid options are:

```
 -C <col>
  Sets the attribute index.
```

```
 -V <index1,index2-index4,...>
  Specify the list of values to indicate. First and last are
  valid indexes (default last)
```

```
 -N <index>
  Set if new boolean attribute nominal.
```

**Version:**
:   $Revision: 5543 $

**Author:**
:   Eibe Frank (eibe@cs.waikato.ac.nz)

**See Also:**: Serialized Form

---

**Constructor Summary**

`MakeIndicator()`
  
          Constructor


| **Method Summary** | |
| --- | --- |
| `java.lang.String` | `attributeIndexTipText()` |
| `java.lang.String` | `getAttributeIndex()`             Get the index of the attribute used. |
| `Capabilities` | `getCapabilities()`             Returns the Capabilities of this filter. |
| `boolean` | `getNumeric()`             Check if new attribute is to be numeric. |
| `java.lang.String[]` | `getOptions()`             Gets the current settings of the filter. |
| `java.lang.String` | `getRevision()`             Returns the revision string. |
| `java.lang.String` | `getValueIndices()`             Get the indices of the indicator values. |
| `Range` | `getValueRange()`             Get the range containing the indicator values. |
| `java.lang.String` | `globalInfo()` |
| `boolean` | `input(Instance instance)`             Input an instance for filtering. |
| `java.util.Enumeration` | `listOptions()`             Returns an enumeration describing the available options. |
| `static void` | `main(java.lang.String[] argv)`             Main method for testing this class. |
| `java.lang.String` | `numericTipText()` |
| `void` | `setAttributeIndex(java.lang.String attIndex)`             Sets index of the attribute used. |
| `boolean` | `setInputFormat(Instances instanceInfo)`             Sets the format of the input instances. |
| `void` | `setNumeric(boolean bool)`             Sets if the new Attribute is to be numeric. |
| `void` | `setOptions(java.lang.String[] options)`             Parses a given list of options. |
| `void` | `setValueIndex(int index)`             Sets index of the indicator value. |
| `void` | `setValueIndices(java.lang.String range)`             Sets indices of the indicator values. |
| `void` | `setValueIndicesArray(int[] indices)`             Set which attributes are to be deleted (or kept if invert is true) |
| `java.lang.String` | `valueIndicesTipText()` |

**Methods inherited from class weka.filters.Filter**

`batchFilterFile, batchFinished, filterFile, getCapabilities, getOutputFormat, isFirstBatchDone, isNewBatch, isOutputFormatDefined, makeCopies, makeCopy, numPendingOutput, output, outputPeek, toString, useFilter, wekaStaticWrapper`

**Methods inherited from class java.lang.Object**

`equals, getClass, hashCode, notify, notifyAll, wait, wait, wait`

**Constructor Detail**

### MakeIndicator

```
public MakeIndicator()
```

:   Constructor


**Method Detail**

### getCapabilities

```
public Capabilities getCapabilities()
```

:   Returns the Capabilities of this filter.

    :   **Specified by:**: `getCapabilities` in interface `CapabilitiesHandler` **Overrides:**: `getCapabilities` in class `Filter`
    :   **Returns:**: the capabilities of this object **See Also:**: `Capabilities`

---


### setInputFormat

```
public boolean setInputFormat(Instances instanceInfo)
                       throws java.lang.Exception
```

:   Sets the format of the input instances.

    :   **Overrides:**: `setInputFormat` in class `Filter`
    :   **Parameters:**: `instanceInfo` - an Instances object containing the input instance structure (any instances contained in the object are ignored - only the structure is required). **Returns:**: true if the outputFormat may be collected immediately **Throws:**: `UnsupportedAttributeTypeException` - the selecte attribute is not nominal: `UnsupportedAttributeTypeException` - the selecte attribute has fewer than two values.: `java.lang.Exception` - if the inputFormat can't be set successfully

---


### input

```
public boolean input(Instance instance)
```

:   Input an instance for filtering. The instance is processed
    and made available for output immediately.

    :   **Overrides:**: `input` in class `Filter`
    :   **Parameters:**: `instance` - the input instance **Returns:**: true if the filtered instance may now be collected with output(). **Throws:**: `java.lang.IllegalStateException` - if no input format has been set.

---


### listOptions

```
public java.util.Enumeration listOptions()
```

:   Returns an enumeration describing the available options.

    :   **Specified by:**: `listOptions` in interface `OptionHandler`
    :   **Returns:**: an enumeration of all the available options.

---


### setOptions

```
public void setOptions(java.lang.String[] options)
                throws java.lang.Exception
```

:   Parses a given list of options.


    Valid options are:

    ```
     -C <col>
      Sets the attribute index.
    ```

    ```
     -V <index1,index2-index4,...>
      Specify the list of values to indicate. First and last are
      valid indexes (default last)
    ```

    ```
     -N <index>
      Set if new boolean attribute nominal.
    ```

    :   **Specified by:**: `setOptions` in interface `OptionHandler`
    :   **Parameters:**: `options` - the list of options as an array of strings **Throws:**: `java.lang.Exception` - if an option is not supported

---


### getOptions

```
public java.lang.String[] getOptions()
```

:   Gets the current settings of the filter.

    :   **Specified by:**: `getOptions` in interface `OptionHandler`
    :   **Returns:**: an array of strings suitable for passing to setOptions

---


### globalInfo

```
public java.lang.String globalInfo()
```

:   **Returns:**: a description of the filter suitable for displaying in the explorer/experimenter gui

---


### attributeIndexTipText

```
public java.lang.String attributeIndexTipText()
```

:   **Returns:**: tip text for this property suitable for displaying in the explorer/experimenter gui

---


### getAttributeIndex

```
public java.lang.String getAttributeIndex()
```

:   Get the index of the attribute used.

    :   **Returns:**: the index of the attribute

---


### setAttributeIndex

```
public void setAttributeIndex(java.lang.String attIndex)
```

:   Sets index of the attribute used.

    :   **Parameters:**: `attIndex` - the index of the attribute

---


### getValueRange

```
public Range getValueRange()
```

:   Get the range containing the indicator values.

    :   **Returns:**: the range containing the indicator values

---


### valueIndicesTipText

```
public java.lang.String valueIndicesTipText()
```

:   **Returns:**: tip text for this property suitable for displaying in the explorer/experimenter gui

---


### getValueIndices

```
public java.lang.String getValueIndices()
```

:   Get the indices of the indicator values.

    :   **Returns:**: the indices of the indicator values

---


### setValueIndices

```
public void setValueIndices(java.lang.String range)
```

:   Sets indices of the indicator values.

    :   **Parameters:**: `range` - the string representation of the indicator value indices **See Also:**: `Range`

---


### setValueIndex

```
public void setValueIndex(int index)
```

:   Sets index of the indicator value.

    :   **Parameters:**: `index` - the index of the indicator value

---


### setValueIndicesArray

```
public void setValueIndicesArray(int[] indices)
```

:   Set which attributes are to be deleted (or kept if invert is true)

    :   **Parameters:**: `indices` - an array containing indexes of attributes to select. Since the array will typically come from a program, attributes are indexed from 0. **Throws:**: `InvalidArgumentException` - if an invalid set of ranges is supplied

---


### numericTipText

```
public java.lang.String numericTipText()
```

:   **Returns:**: tip text for this property suitable for displaying in the explorer/experimenter gui

---


### setNumeric

```
public void setNumeric(boolean bool)
```

:   Sets if the new Attribute is to be numeric.

    :   **Parameters:**: `bool` - true if new Attribute is to be numeric

---


### getNumeric

```
public boolean getNumeric()
```

:   Check if new attribute is to be numeric.

    :   **Returns:**: true if new attribute is to be numeric

---


### getRevision

```
public java.lang.String getRevision()
```

:   Returns the revision string.

    :   **Specified by:**: `getRevision` in interface `RevisionHandler` **Overrides:**: `getRevision` in class `Filter`
    :   **Returns:**: the revision

---


### main

```
public static void main(java.lang.String[] argv)
```

:   Main method for testing this class.

    :   **Parameters:**: `argv` - should contain arguments to the filter: use -h for help


---


|  |  |  |
| --- | --- | --- |
| **Overview** \| **Package** \| **Class** \| **Tree** \| **Deprecated** \| **Index** \| **Help** \| **Weka's home** | |  |
| **PREV CLASS**   **NEXT CLASS** | **FRAMES**    **NO FRAMES**     **All Classes** |  |
| SUMMARY: NESTED \| FIELD \| CONSTR \| METHOD | DETAIL: FIELD \| CONSTR \| METHOD |  |


---
